# Utilization Focused Evaluation at Bahria University Medical & Dental College

**DOI:** 10.12669/pjms.334.13020

**Published:** 2017

**Authors:** Rehana Rehman, Rabiya Ali, Hina Moazzam, Saifullah Shaikh

**Affiliations:** 1 Dr. Rehana Rehman, MBBS, M.Phil, Ph.D. Assistant Professor, Department of Biological & Biomedical Sciences, Agha Khan University Hospital, Karachi, Pakistan; 2 Dr. Rabiya Ali, MBBS. MPhil/Ph.D Scholar Senior Lecturer, Department of Physiology, Bahria University Medical & Dental College, Karachi, Pakistan; 3 Dr. Hina Moazzam, MBBS, MPhil Scholar Senior Lecturer, Department of Physiology, Bahria University Medical & Dental College, Karachi, Pakistan; 4 Dr. Saifullah Shaikh, MBBS, M.Sc., D.Diab Assistant Professor, Department of Physiology, Bahria University Medical & Dental College, Karachi, Pakistan

**Keywords:** Utilization Focused Evaluation (UFE), Program Evaluation, Primary Intended Users (PIU), Neuroscience Module

## Abstract

**Objective:**

To evaluate integrated learning program of neurosciences for continuation of integrated learning in the forthcoming teaching and learning modules of undergraduate medical curriculum at Bahria University Medical & Dental College (BUMDC).

**Methods:**

A mixed method design was conducted from August 2016to February2017 after ethical approval from BUMDC. The quantitative aspect was evaluated retrospectively by desk records ofmarks obtained in integrated module and nonintegrated module. Focused group discussionwere conducted with primary intended users (chair of integration committee, faculty and students of first and second year MBBS)to share their expectations and concerns and get responses on key evaluation questions for implementationand outcome evaluation of integrated learning program.

**Results:**

The desk record revealed a positive perception of students and faculty at the time of implementation with improvement in results after integration in subjects of basic sciences. The discussions highlighted reasons which resulted in failure of its continuation and affirmedreadiness for re-induction and continuation of integration with clinicalsciences.

**Conclusion:**

Evaluators considered approval and re-application of integrated curriculum at BUMDC after utilization focused evaluation.

## INTRODUCTION

The conventional non-integrated approach of MBBS curriculum disseminates knowledge by afragmented approach which fails to build learning skills of case investigations and analysis. As a result of which students fail to acquire conceptual understanding of the topic and hence its application in treatment and prevention of disease.^1^ A number of integrated approaches have been put forward in medical curricula to ensure holistic approach required for meaningful learning.^2^,^3^

In Bahria University Medical & Dental College (BUMDC) teaching of basic science subjects is accomplished by modular and hybrid system with a mixture of traditional teaching and problem based learning (PBL). Integrated Learning Program (ILP) was implemented in 2010 to integrate the subjects of Anatomy, Physiology, Biochemistry, and Community Medicine disciplines in module of Neurosciences.^1^

Although, ILP was executed with favorable results, yet integration was not followed in the succeeding modules. With this the stake holders taught about its evaluation by external evaluators with the aim to ascertain its usefulness and efficacy in real time environment. They wanted to identify any gaps, deficiencies and weakness for implementation and improvement in the forthcoming modules. In order to acquire this objective, utilization focused evaluation (UFE) with its 17 steps framework^4^,^5^ was applied to evaluate ILP at BUMDC.

## METHODS

**Fig. 1:**
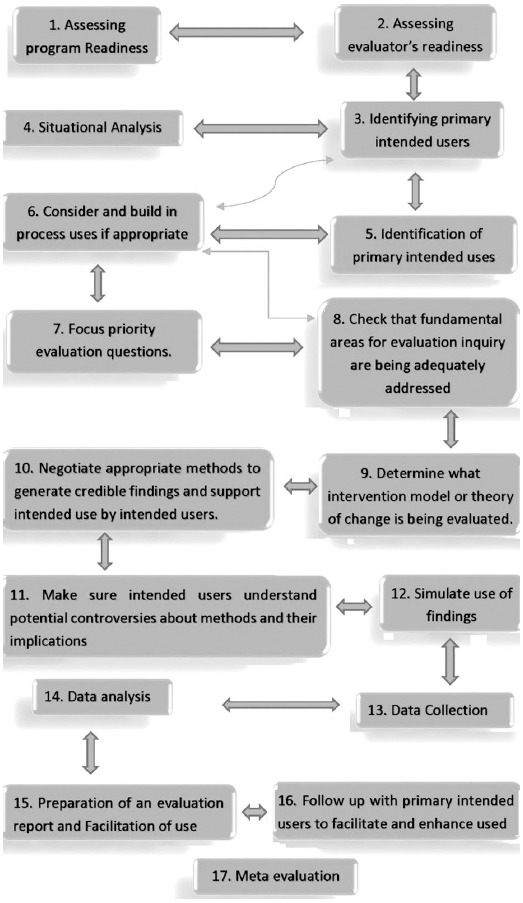
Diagram of the UFE steps.

The study was conducted from August 2016 to February 2017 after ethical approval from BUMDC. A mixed method design was adopted with the qualitative aspect acquired by Focused group discussion (FGD) and quantitative data was assessed retrospectively by the available desk records from January 2012 to December 2015. The evaluator team comprised of external and internal evaluators selected on the basis of their command on professional practical knowledge, systemic enquiry skills, project management skills, reflective practice competence and interpersonal competence from inside and outside the institution. To assess and enhance readiness for evaluation, evaluators shared their expectations and concerns with the primary intended users (PIU); chair integration committee, students, teachers from basic and clinical sciences. Evaluation of ILP was done by all the steps of UFE as shown in Fig. 1. The evaluators built the process of evaluation in terms of goal of the program, organizational support and limitations, apprehensions and restraints and feedback from PIU. They formulated the key evaluation questions (KEQ) (Table-I) after listening to priority concerns of PIU.^6^

**Table-I:** Key Evaluation Questions (KEQ) for evaluation of Program.

	*Key Evaluation Questions*	
Implementation Evaluation	Were there intra and interdepartmental discussions before implementation of ILP?	

	Was the framing of time table aligned with objectives?	

	Was integrated contents delivered to students by guide book?	

	Was the assessment of students integrated in theory and practical examination?	

	Were the teachers reluctant to adopt the change?	

Outcome Evaluation	Reaction	How did the students respond to the program?
		How did faculty respond to the program?
		Does faculty think that they felt overworked?

	Learning	Did learners enjoy the way contents were delivered?
		Do you think knowledge transfer was better than in non-integrated modules?
		Were students able to integrate basic science knowledge in health and disease?

	Behavior	Did ILP help to develop positive attitude towards medical education in faculty members?
		Did the program develop coherence and acceptance among different faculty members
		Did students develop communication skills as a result of implementation?
		Was the program cost effective?
		Was there a change in psychomotor skills at the end of the program?

	Result	Was there any improvement in performance of students in module examinations with reference to NI module?
		Will program improve University exam results?
		Will it assist their clinical orientation and medical practice?

Three focus group discussions (FGD) with; chair of integration committee, faculty members and students were conducted by KEQ developed by the researchers from an iterative literature process. All the faculty members of Basic Sciences especially those who took part in integration of Neuroscience Module (2010) were invited. Medical students of first and second year MBBS were invited and informed about the purpose of FGD. They were later short listed on the basis of their academic performance (scores in the previous modules). The evaluators took consent from the participants and confirmed about anonymity and confidentiality of data. Each FGD lasted for approximately 60to 90 minutes in a private place free from noise and disturbance and were audio taped after obtaining the consent. The data obtained after debriefing from FGD answered all KEQs in terms of difficulties faced during implementation of the program, perception of usefulness of program by students and teachers. The quantitative data of results of nonintegrated (NI) and integrated module after ILP, guide book for students, schedule of both modules, feedback forms filled by students and faculty members was also acquired and analyzed. The comparative analysis of modules done in SPSS-15 with comparison by Student’s t -test was taken into account.

## RESULTS

### Quantitative Results

On the basis of retrieved desk records, comparison of module results after intervention of ILP declared better performance of medical students after integration. The upgrading of results attained by ILP in disciplines of Anatomy, Physiology and Biochemistry in neuroscience module. In discipline of Anatomy interactive sessions and model study facilitated in understanding of structural and functional association of nervous system in 86% students through reinforcement, interpretation and description of structured objectives in pleasant manner. Assimilation of knowledge in 80.25% of students was the result of interactive lectures of biochemistry linked with molecular and functional aspects. Elucidation of physiological mechanisms that construct basis of disease assisted 84% of students to grasp pertinent pathologies. Implementation of ILP apprehended students greater expertise and interest on subject and effectiveness of integrated teaching for better scoring in assessments and clinical postings. The feedback response from students and faculty suggested that problem solving skills, through provision of structured integrated model of neurosciences was made possible.

Qualitative Results of qualitative analysis was provided after thematic analysis from responses to all the KEQ, sorted out in the form of usefulness of ILP and steps required for outcome and implementation evaluation. One of the faculty members responded; “Brain Storming, Self learning, Connections among all subjects and a sense of accomplishment was acquired by the students during ILP”. “Framing of time table” was the most important impediment to its continuation, mentioned by few participants. A senior faculty member mentioned; “Interpersonal skills in terms of listening, and receiving criticism developed positive attitude, when we implemented ILP way back in 2010”. One of the student said; “we lack integration of basic sciences in clinical practice that needs to be catered by implementation of case based session”. Majority of students in FGD recommended that understanding and application of Basic Sciences in clinical scenarios can be improved by integration of modules. The chair said; “we were not able to take forward integrated learning due to the reason that all the schedules were not made after intra and interdepartmental discussions among “Basis Science Departments” (Anatomy, Physiology, Biochemistry, and Community Medicine) with contribution from clinical faculty”. Furthermore, he said “Objectives were not aligned with the learning outcome and mode of information transfer hence integration could not be achieved “Faculty criticized that the time table of whole module was not finalized before the start of the module rather was constructed after it had started which resulted in serious flaws. In the FGD with chair of integration committee, it was concluded that since all the political powers are in favor of implementation of integration, all efforts should be made to make it possible. The discussions with stake holders on the basis of KEQ (Table-I) thus showed readiness after outcome evaluation for implementation and sustainability of the program. Moreover there were suggestions to finalize the guide book and disseminate it before start of the session.

## DISCUSSION

Program evaluation is recognition, purification and implementation of secure benchmarks to govern the objectives of evaluation meaningfully. The goal of evaluation was focused on organization of central themes or concepts that combined several subjects using multiple learning strategies in ILP.^7^,^8^ The mission was accomplished by evaluating objectives of all subjects integrated in each module and implemented first of all in the ongoing (neuroscience) module.^9^

The two imperative hypothesis of UFE are that no evaluation should progressunless there are managers who will essentially be working on proofs that the evaluation will engender and dynamically participated in the course of overall assessment.^10^ In UFE, tactics and data assembling tools are selected on the basis of the KEQs that are suggested from the ‘users’.^4^ Principal strategic concern in UFE is the planned utilization of outcomes to enlighten perspective outlines instead of the improvement of understanding.^11^,^12^

The evaluation model used at BUMDC connected all stakeholders in planning of evaluation, given responses and understanding of findings.^13^ We gathered central decision-makers (evaluators and organization) at one platform, identified their data requirements and highlighted the envisioned usage of the results and policy-making). On the basis of context of change required to improve the program.^4^,^13^ The objective of including stakeholders was on the basis of making decisions for effective development, transition and transformation of the program^14^,^15^ so that, intended users are more likely to understand and use evaluations by active involvement in the process.^14^,^15^

UFE determines practical agenda for conducting evaluations with progress of its utility.^16^ The approach used by our evaluators is similar to evaluation of a psychological health program, Youth Net/Réseau Adolescents (YN/RA), that has served as a classic model to achieve perpetual advancement in skilled training and aftereffects for adolescence.^13^ The approach was particularly selected to effectuate a youth-friendly evaluation through stakeholder oriented course modifications. Thus, the YN/RA evaluation primarily signifies an essential progress in the assessment of youth mental health policies and services.^13^ UFE tactics were also introduced to evaluate objective structured clinical skills evaluation (OSCE) program that were used in the new Bachelor of Science(BSC). In General and Psychiatric Nursing Registration agenda presented in an Institute of Technology in the south west of Ireland.^12^ UFE was used for development of social, and financial curriculum for youth by afflation. The findings proposed by evaluators helped in consideration of organizational and operational decisions for development of program.^4^

The application of UFE actively involved PIU in the decision making process with the guidance for improvement of program. The most important limitation was time interval in application of program and its evaluation however the approach of UFE can look up for the causes of failure of its continuation. It would further give emphasis to solve the problems with well-defined goals and strategic directions acquired by feedback from all PIU.

## CONCLUSION

Evaluators considered approval and application of transformation from nonintegrated to integrated curriculum at BUMDC in view of recommendations from chair integration committee, faculty members and students and desk record of an integrated module. The evaluators also highlighted the benefits of integration in undergraduate curriculum and informed them that any insufficiencies at the end of evaluation will help in further modification in the forthcoming modules.

### Recommendations

Organizations should employ UFE^14^ to develop coordinated efforts and bring transitions from traditional to unidisciplinary and finally multidisciplinary curriculum for effective learning.

### Authors’ Contribution

***Rehana Rehman:*** Principal Investigator took part in conception and study design & compilation of write up, drafting the article & revising it critically for important intellectual content.

***Rabiya Ali:*** Took part in study design, acquisition, analysis and interpretation of data, compilation of write up drafting the article.

***Hina Moazzam:*** Took part in compilation of write up and formulation of tables.

***Saifullah Shaikh:*** Took part in data analysis.

***Rabiya Ali:*** Takes the responsibility and is accountable for all aspects of the work in ensuring that questions related to the accuracy or integrity of any part of the work are appropriately investigated and resolved.

## References

[1] Rehman R, Iqbal A, Syed S, Kamran A (2011). Evaluation of integrated learning program of undergraduate medical students. Pak J Physiol.

[2] Shimura T, Aramaki T, Shimizu K, Miyashita T, Adachi K, Teramoto A (2004). Implementation of integrated medical curriculum in Japanese medical schools. J Nippon Med School.

[3] Rafique N (2014). Designing and implementation of vertically and horizontally integrated endocrinology and reproduction module. Pak J Physiol.

[4] Ramirez R, Kora G, Shephard D (2015). Utilization Focused Developmental Evaluation: Learning Through Practice. J Multidisciplinary Evaluation.

[5] Patton MQ, Knight V, Connelly S (2008). Evaluation worth using: Utilization Focused Methods Decisions, Utilization-focused evaluation.

[6] Clark H, Anderson AA (2004). Theories of change and logic models: Telling them apart. American Evaluation Association Conference.

[7] Domik G, Goetz F (2006). A Breadth-First Approach for Teaching Computer Graphics. Eurographics (Education Papers).

[8] Kate MS, Kulkarni UJ, Supe A, Deshmukh YA (2010). Introducing integrated teaching in undergraduate medical curriculum. Int J Pharm Sci Res.

[9] Fitzpatrick JL, Sanders JR, Worthen BR (2004). Objectives-Oriented Evaluation Approaches, Program evaluation: Alternative approaches and practical guidelines.

[10] Ramírez R, Brodhead D, Solomon C, Kumar-Range S, Zaveri S, Earl S, Smith M (2013). The DECI project, Utilization focused evaluation: A primer for evaluators.

[11] Flowers AB (2010). Blazing an evaluation pathway: Lessons learned from applying utilization-focused evaluation to a conservation education program. Evaluation Program Planning.

[12] Brosnan M, Evans W, Brosnan E, Brown G (2006). Implementing objective structured clinical skills evaluation (OSCE) in nurse registration programmes in a centre in Ireland: A utilization focused evaluation. Nurse Educ Today.

[13] Armstrong LL (2009). A utilization-focused approach to evaluating a “youth-friendly” mental health program: The Youth Net/Réseau Ado story. Vulnerable Children and Youth Studies.

[14] Vassar M, Wheeler DL, Davison M, Franklin J (2010). Program evaluation in medical education: An overview of the utilization-focused approach. J Educ Eval Health Prof.

[15] Patton MQ, Knight V, Habib L (2011). Make sure intended users understand potential methods controversies and their implications. Essentials of Utilization-Focused Evaluation.

[16] Solomon C, Earl S, Hay KE, Kumar-Range (2014). An Evaluation Practitioner’s Journey with Utilization-focused Evaluation, Making Evaluation Matter. Writings from South Asia IDRC.

